# Modified Western blotting for insulin and other diabetes-associated peptide hormones

**DOI:** 10.1038/s41598-017-04456-4

**Published:** 2017-07-31

**Authors:** Naoyuki Okita, Yoshikazu Higami, Fumio Fukai, Masaki Kobayashi, Miku Mitarai, Takao Sekiya, Takashi Sasaki

**Affiliations:** 1 Department of Molecular Metabolic Regulation Research, Sasaki Institute, Sasaki Foundation, 2-2 Kandasurugadai, Chiyoda-ku Tokyo, 101-0062 Japan; 2 Translational Research Center, Research Institute for Science and Technology, Tokyo University of Science, 2641 Yamazaki, Noda Chiba, 278-0022 Japan; 3 Laboratory of Molecular Pathology and Metabolic Disease, Faculty of Pharmaceutical Sciences, Tokyo University of Science, 2641 Yamazaki, Noda Chiba, 278-0022 Japan; 4 Laboratory of Molecular Patho-Physiology, Faculty of Pharmaceutical Sciences, Tokyo University of Science, Chiba, 278-0022 Japan; 5 Department of Cancer Genome Research, Sasaki Institute, Sasaki Foundation, 2-2 Kandasurugadai, Chiyoda-ku Tokyo, 101-0062 Japan; 6 Division of Translational and Molecular Medicine, Research Center for Medical Sciences, The Jikei University School of Medicine, 163-1 Kashiwashita, Kashiwa Chiba, 277-8567 Japan

## Abstract

Now, the quantification of proinsulin/insulin contents within organisms tends to be evaluated only by enzyme-linked immunosorbent assay (ELISA), although assessing the adequacy of results by some quantification method is important. Remarkably, few scientific papers use detection by Western blotting (WB), another immunological assay, of proinsulin/insulin. We found two problems with quantification of insulin and proinsulin by general WB: the shape of an insulin band in gel electrophoresis is distorted, and the retention potency to a blotting membrane of the peptide hormones (mainly insulin) is low. We solved the first problem by optimizing the sodium dodecyl sulfate concentration in the sample buffer and the second problem by glutaraldehyde fixation following treatment with a blocking solution for a short time. The improvements were confirmed by quantification of proinsulin/insulin in standards, MIN6c4 cell lysates, and MIN6c4 culture supernatants. Furthermore, we showed that the modified WB is applicable to other diabetes-associated peptide hormones: insulin analogs, glucagon, GLP-1s, somatostatins, ghrelins, and pancreatic polypeptide. Our data showed that the modified WB can contribute to qualitative or quantitative analyses of diabetes-associated peptides by providing analytical information based on electrophoresis, although ELISA, which is an almost exclusive method in the quantification of peptide hormones, supplies only numerical data.

## Introduction

As the pathogenesis of diabetes is closely associated with pancreatic peptide hormones including insulin, quantitative analysis of their intra- and intercellular contents is important in the contexts of diagnosis, therapy, and research of diabetes^1^. Insulin (51 a.a., 5807.6 Da) is composed of an A-chain (21 a.a., 2383.7 Da) and a B-chain (30 a.a., 3429.9 Da) and is the only hormone to possess a direct hypoglycemic action. The process of insulin maturation has been well-characterized^1, 2^. Insulin is transcribed as preproinsulin, a precursor single polypeptide, from the INS gene and is transported into the endoplasmic reticulum by the action of the N-terminal signal sequence. Preproinsulin is converted to proinsulin (86 a.a., 9394.7 Da) by cleavage of the signal sequence during the transport process. Endoplasmic reticulum-localized proinsulin is properly folded by the formation of disulfide bonds: two inter-chain (between the A-chain and the B-chain) disulfide bridges at cysteine residues, and one intra-chain (in the A-chain) disulfide bridge at cysteine residues. Finally, C-peptide is cleaved out from proinsulin by several prohormone convertases (PC1/3, PC2, and CPE) in the Golgi apparatus, and then maturation to insulin is completed.

Enzyme-linked immunosorbent assay (ELISA) is used almost exclusively in the quantification of insulin and is an excellent method for the quantification of target proteins. However, one must constantly be concerned about specificity because we cannot obtain any information other than the raw data in the analysis. In the case in which the target is a peptide hormone, immunological cross-reaction between the prohormone and the mature hormone is also an important problem. In the area of diabetes research, not only insulin but also glucagon is one of the molecules that is difficult to quantify with ELISA^3^. Western blotting (WB) is a semi-quantitative technique widely used in protein analysis. However, we questioned whether WB of insulin is applicable because there are only a few scientific papers on the use of WB to detect insulin. When we actually confirmed the usability of WB in insulin detection, we found that there are some problems in the quantitative analysis of insulin by WB, including distortion of insulin signal bands and the lower sensitivity of insulin but not of proinsulin. In the present study, we clarified the reasons why the detection of insulin and proinsulin by WB is not used widely and then succeeded in resolving these problems by altering the general protocol of WB. Additionally, we showed the usability of the improved WB for the detection of other diabetes-associated peptide hormones in various biological samples.

## Results

### Optimization of SDS concentration in Tris/Tricine/urea SDS-PAGE system for insulin detection

**Figure 1:**
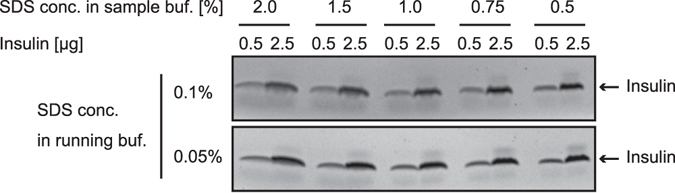
Optimization of SDS concentration in Tris/Tricine/urea SDS-PAGE system for insulin detection. Reference standards of insulin (0.5 or 2.5 µg) were prepared by incubation with the SDS sample buffers containing the indicated concentration of SDS and subjected to Tris/Tricine/urea SDS-PAGE using the running buffer containing 0.1% or 0.05% SDS. The gels were stained with CBB R-250.

It is known that the Tris/Tricine/urea sodium dodecyl sulfate polyacrylamide gel electrophoresis (SDS-PAGE) system is an appropriate method for use in the gel electrophoresis of peptide hormones^4^. However, we noticed that insulin bands in WB using the original protocol of Tris/Tricine/urea SDS-PAGE are distorted and streaked. We wondered whether the SDS concentration in the running buffer or the sample buffer is too high to detect small peptide hormones such as insulin because the original paper referred to the effects of SDS on such phenomenon^4^. Hence, we sought to determine the optimized condition. As shown in Fig. 1, decreasing the SDS concentration in both buffers contributes to the formation of a sharp insulin band. We judged that an SDS concentration of 0.05% is better in the running buffer. With regard to the optimized SDS concentration in the sample buffer, after considering that 1.4 g SDS binds to 1 g protein, we adopted a value of 1%: this concentration of SDS can deal with a protein solution to approximately 7 µg/µL.

### During the general WB method, insulin is easily washed out from the blotted membrane compared with proinsulin

**Figure 2:**
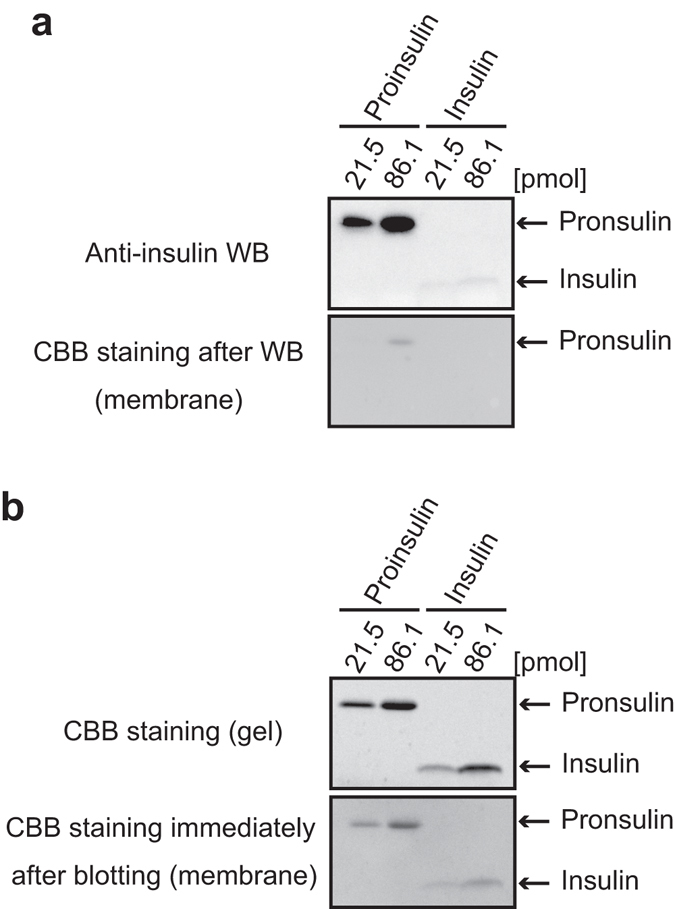
During the general WB method, insulin is easily washed out from the blotted membrane compared with proinsulin. The indicated doses of proinsulin and insulin were separated by Tris/Tricine/urea SDS-PAGE. (**a**) WB was performed by using anti-insulin clone c27c9, which recognizes both proinsulin and insulin (upper panel). After WB detection, the membrane was stained with CBB R-250 (lower panel). In the condition, insulin was below detection limit of CBB staining of the membrane. (**b**) The PVDF membrane immediately after electro-blotting for WB (upper panel) was stained with CBB R-250. As another experiment, the separated gel was directly stained with CBB R-250 (lower panel).

Other than the insulin band shape, we also faced a problem in improving insulin quantification. The problem is that the WB signal of insulin is weaker than that of proinsulin, even though we analyzed equal moles of proinsulin/insulin with a monoclonal antibody (anti-insulin clone c27c9) that can recognize both proinsulin and insulin (Fig. 2a, upper panel). Coomassie brilliant blue (CBB) staining of the polyvinylidene difluoride (PVDF) membrane after WB detection showed that proinsulin, but not insulin, was retained on the PVDF membrane after WB (Fig. 2a, lower panel). We considered two possibilities as the cause: detachment of insulin from the PVDF membrane during the WB procedure after electro-blotting or trouble in capturing the insulin onto the PVDF membrane. As shown in Fig. 2b, we were able to confirm the existence of insulin protein on the membrane subjected to CBB staining immediately after electro-blotting. These results indicate that only insulin is washed out from the blotted PVDF membrane during the standard WB procedure.

### Glutaraldehyde efficiently fixes insulin on the blotted membrane

**Figure 3:**
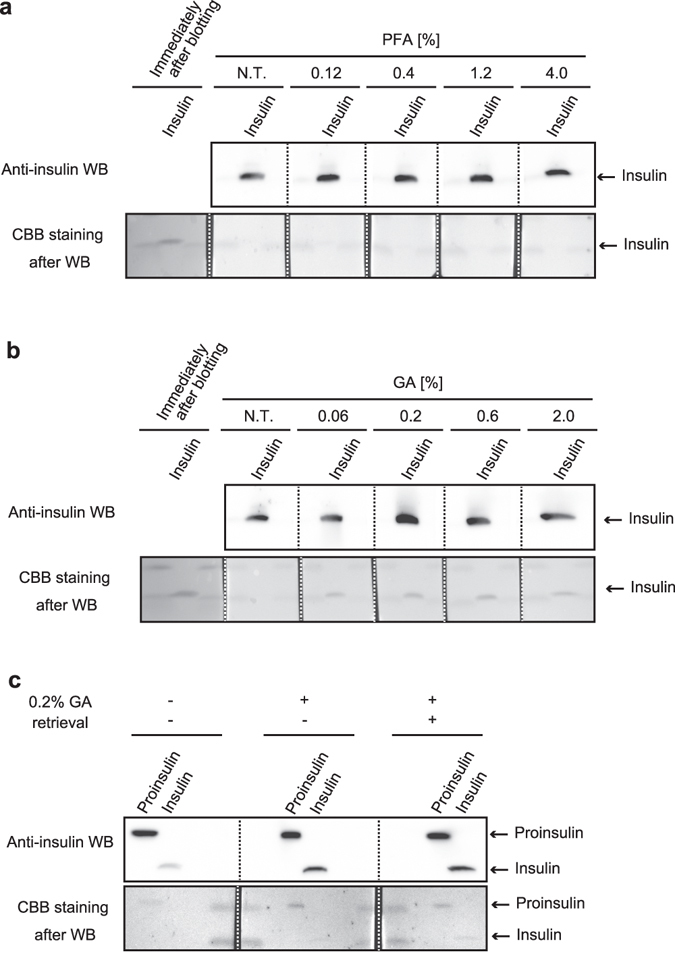
Glutaraldehyde efficiently fixes insulin on the blotted membrane. (**a,b**) Reference standard of insulin (1 µg) was subjected to Tris/Tricine/urea SDS-PAGE. After electro-blotting, the one blotted membrane was separated into 1 slip for the control experiment (Coomassie brilliant blue [CBB] staining immediately after electro-blotting, labeled as “Immediately after blotting”) and 5 slips for the aldehyde treatment. Paraformaldehyde (PFA) (**a**) or glutaraldehyde (GA) (**b**) concentrations were verified as shown. N.T. indicates a control treatment. (**c**) Reference standards of proinsulin (200 ng) and insulin (125 ng) were subjected to Tris/Tricine/urea SDS-PAGE. After electro-blotting, the blotted membrane was separated into 3 slips to verify the effect of the retrieval step on insulin Western blotting (WB). Upper panels are the results of WB of insulin, and the lower panels are the results of CBB staining of the blotted membrane. To compare the effects of various conditions, WB images in each sub-part of the figure were captured at one time.

To resolve the “wash-out problem,” we considered applying optional WB procedures for efficiently fixing a target protein onto a blotted membrane: heating^5^, alkylating treatment^6^, and aldehydes treatment^7–10^. We precisely examined the optimized condition of aldehyde treatment on the retention of insulin onto a PVDF membrane because preliminary experiments showed that aldehyde treatment would lessen the problem. After the electro-blotting step in the WB procedure, the blotted membrane was treated with various concentrations of paraformaldehyde (PFA) (Fig. 3a) or glutaraldehyde (GA) (Fig. 3b), and then the blocking step was pursued. As a result, GA but not PFA treatment increased the signal of the insulin WB. Considering that CBB-stained insulin on the blotted membrane was recovered by treatment with GA (Fig. 3b, lower panel), the result mean that GA fixes insulin on the blotted membrane, resulting in recovery of the insulin WB signal. The optimum concentration of GA was 0.2%, and concentrations greater than 0.2% tended to exhibit an inhibitory effect on the signal. We thought that too much cross-linkage by aldehyde fixation caused such a phenomenon as is often observed in immunohistochemical and immunocytochemical studies. Therefore, we also examined the usability of the other immunohistochemical procedures such as antigen retrieval and quenching steps in insulin WB (Fig. 3c). The result showed that the retrieval step by citrate buffer slightly enhanced the signal of both insulin and proinsulin. As an important caution, the glycine-quenching step should be performed after, but not before, the retrieval step (Supplemental Fig. 1).

### Treatment with blocking buffer in pre-fixed blots contributes to the improvement of WB signals from insulin

**Figure 4:**
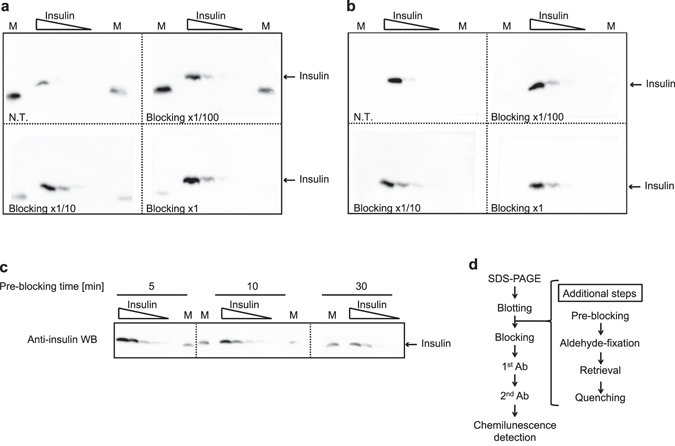
Treatment of blocking buffer in pre-fixed blots enhances the improvement of WB signals from insulin. (**a,b**) Dilution series (panel a for low doses: 10, 3, 1, 0.3 ng and panel b for high doses: 100, 30, 10, 3 ng) of insulin were subjected to Tris/Tricine/urea SDS-PAGE. M indicates molecular weight markers. For concentration evaluation, 4 slips from the one blotted membrane were treated with the indicated concentration of blocking solution. (**c**) For time optimization, the dilution series (10, 3, 1, 0.3, 0.1 ng) of insulin were subjected to Tris/Tricine/urea SDS-PAGE. Three slips from the one blotted membrane were treated with the 1x blocking solution for the indicated time. M indicates a lane for molecular weight markers. One of the containing proteins cross-reacts with anti-insulin antibodies. (**d**) Flow diagram of the improved proinsulin/insulin WB. To compare the effects of the various conditions, WB images in each sub-part of the figure were captured at one time.

When evaluating the usability of the GA-fixing method, we noticed that the effect of GA treatment on insulin retention tended to decrease in the case of the analysis of a low content (a few ng) of insulin. Therefore, we thought that the provision of scaffold proteins for cross-linkage onto a PVDF membrane would be able to resolve this problem. For this purpose, the use of a blocking solution containing various proteins seemed to be appropriate. Briefly, we sought to try a “pre-blocking treatment” by soaking a blotted membrane with a blocking solution for 30 min before GA fixation. The signal intensity was enhanced in accord with the concentration of the blocking solution in WB of a low content of insulin (10 ng to 0.3 ng) (Fig. 4a), whereas none of the doses of the solution affected the signal strength significantly in WB of higher contents of insulin (100 ng to 3 ng) (Fig. 4b). Furthermore, the enhancement effect of the pre-blocking treatment on signal intensity was effectively achieved by a shorter time exposure (5 min) of the blocking solution (Fig. 4c). The flow diagram of the improved proinsulin/insulin WB is summarized in Fig. 4d.

### Quantification of insulin content of a mouse β cell line MIN6c4

**Figure 5:**
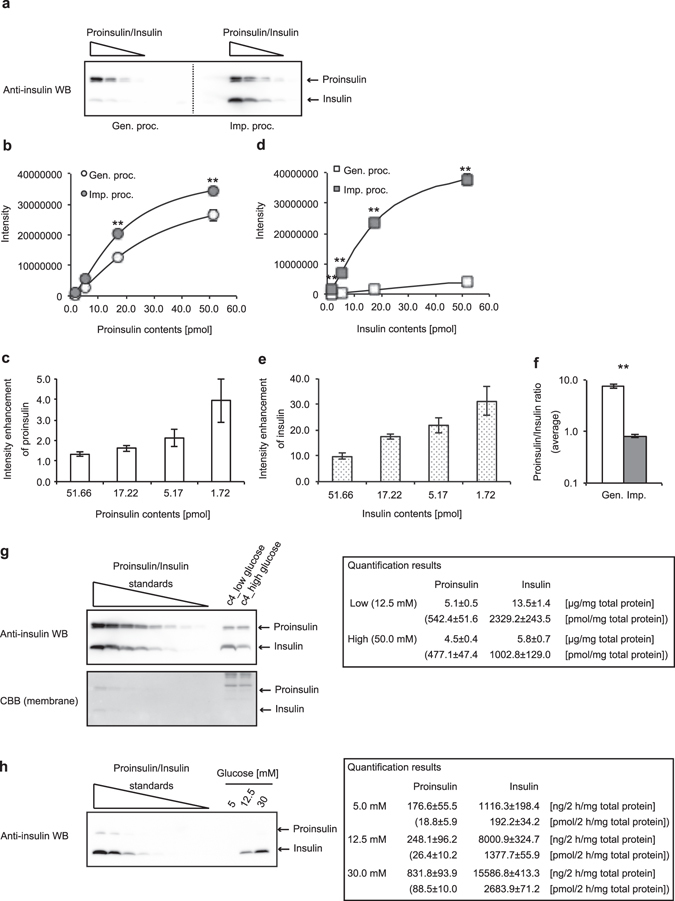
Quantification of insulin contents of a mouse β cell line MIN6c4. Dilution series of equal moles of proinsulin and insulin (range, 51.7 to 1.7 pmol) were subjected to the general (Gen.) or the improved (Imp.) Western blotting (WB) procedure (proc.). The blotted slips for the general or improved protocol were separated from one blotted membrane. (**a**) A representative image from four independent experiments is shown. (**b–e**) The quantification results of proinsulin (**b** and **c**) and insulin (**d** and **e**) were obtained from each of the four slips. The data are shown as mean ± S.E. and were analyzed with a Student *t*-test (**p < 0.01). The calibration curves were obtained by four-parameter logistic fitting methods (**b** and **d**). At each four-plotted point quantified, the enhancement of proinsulin or insulin intensity from the improved procedure against that from the general procedure is shown (**c** and **e**). (**f**) Proinsulin/insulin molar ratios (PI/I ratios) obtained from the general or improved WB are shown as the average from a PI/I ratio at each dose quantified. The data are shown as mean ± S.E. and were analyzed with a Student *t*-test (**p < 0.01). (**g**) Proinsulin and insulin contents of MIN6c4 cultured under the indicated conditions were calculated from the reference curve (dose range, 51.7 to 0.8 pmol) (n = 4). (**h**) Proinsulin and insulin contents secreted from MIN6c4 cells under the indicated conditions were calculated from the reference curve (dose ranges: proinsulin, 215.2 to 3.4 fmol; insulin, 2152.4 to 33.6 fmol) (n = 4).

Subsequently, we compared the quantitative results of proinsulin/insulin WB from the improved method with those of the general method (Fig. 5a–f). To address this issue, we subjected an equimolar amount of proinsulin and insulin per one lane in the electrophoresis because the theoretical signal intensities from both proteins under the same condition are equal. Correlation coefficients of all four calibration curves calculated by a four-parameter logistic fitting method were approximately 1 (Fig. 5b,d). Compared with the general method, the improved method enhanced proinsulin signals by 1.3- to 3.9-fold (Fig. 5c) and insulin signals by approximately 9.8- to 31.2-fold (Fig. 5e). As expected, the proinsulin/insulin ratio calculated from these results also improved to approximately 1, which is the theoretical value (Fig. 5f). These results indicate that the effect of signal enhancement on insulin WB is stronger than that on proinsulin WB and that there is no difference in the reliability of quantification by each method. Furthermore, we confirmed that the enhanced effect was observed in the case in which another anti-insulin antibody that can recognize both proinsulin and insulin was used (Supplemental Fig. 2).

Because usability of the improved WB with the reference standards of proinsulin and/or insulin was shown as indicated above, we sought to validate whether our method is applicable for crude protein samples such as cell lysates, culture supernatants, and serums. For a cell lysate sample, we prepared MIN6c4 cells, a mouse β cell line, which were constantly cultured under a low glucose (12.5 mM) or high glucose (50 mM) condition, and quantified the intracellular levels of proinsulin and insulin by WB (Fig. 5e). The quantification results of proinsulin and insulin were, respectively, 5.1 and 13.5 µg/mg total protein under low glucose and 4.5 and 5.8 µg/mg total protein under high glucose. Considering that the research group that established the cell line reported that the insulin content of MIN6c4 cells cultured with 25 mM glucose is approximately 6 to 8 µg/mg total protein in an ELISA assay^11^, we concluded that the obtained results are appropriate. Next, for a culture supernatant, we prepared glucose-stimulated insulin secretion assay samples (Fig. 5f). The quantification results of proinsulin and insulin were, respectively, 176.6 and 1116.3 ng/2 h/mg total protein at 5 mM glucose stimulation, 248.1 and 8000.9 ng/2 h/mg total protein at 12.5 mM glucose stimulation, and 831.8 and 15586.8 ng/2 h/mg total protein at 30 mM glucose stimulation. Because the group that established the cell line reported that insulin secretions from MIN6c4 cells stimulated with 3 or 25 mM glucose were approximately 200–500 or 1500 to 4000 ng/h/mg total protein, respectively^11^, we judged that the obtained results are acceptable. Unfortunately, when we analyzed serum samples from lean or obese mice under an ad libitum condition, we could not detect any chemiluminescent signals of insulin from the samples (Supp. Fig. 3). The results also showed that the detection limit of proinsulin and insulin with the experimental equipment and reagents that we currently possess is approximately 15 fmol. Next, to further ensure the quantitative ability of the method, we also performed a spike and recovery experiment (Supp. Fig. 4). As shown in the results, the recovery rate in two sample forms (a cell lysate and a cell culture supernatant) was approximately 105–126%. Taken together with these results, we concluded that quantification of proinsulin and insulin by the improved WB is adequately applicable.

### Application for other peptide hormones and analogs involved in diabetes

**Figure 6:**
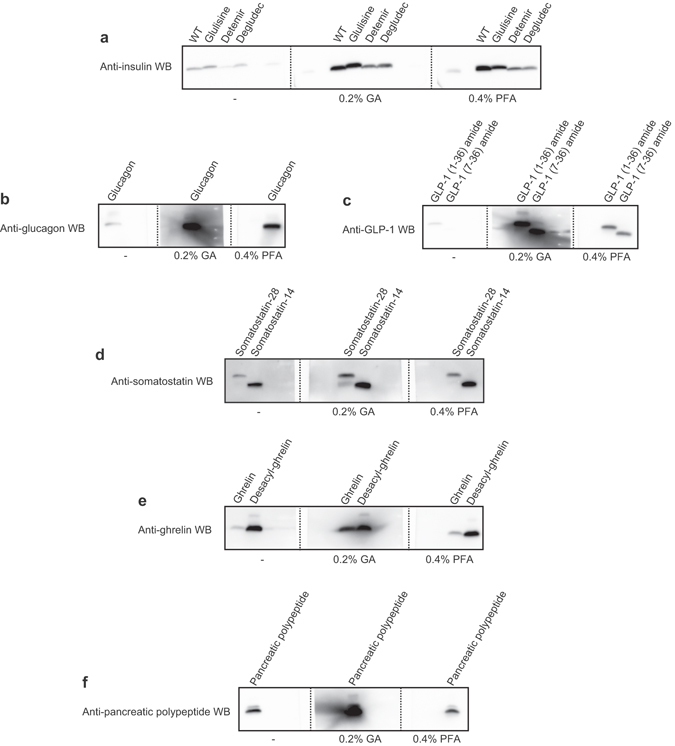
Application for insulin analogs and other peptide hormones involved in diabetes. Reference standard (100 ng) of each peptide was subjected to the improved WB using 0.2% GA or 0.4% PFA as the step of aldehyde treatment or general WB for control. (**a**) Insulin analogs (insulin [wild type], insulin glulisine, insulin detemir, and insulin degludec), (**b**) glucagon, (**c**) GLP-1s (GLP-1 (1-36) amide and GLP-1 (7-36) amide), (**d**) somatostatins (somatostatin-28 and somatostatin-14), (**e**) ghrelins (ghrelin and desacyl ghrelin), and (**f**) pancreatic polypeptide. To compare the effects of various conditions, WB images in each sub-part of the figure were captured at one time.

Because the main purpose of our research group is elucidation of the pathophysiology of diabetes, we evaluated whether the improved WB is applicable for other peptide hormones and analogs involved in diabetes (Fig. 6a, insulin analogs; Fig. 6b, glucagon; Fig. 6c, GLP1s; Fig. 6d, somatostatins; Fig. 6e, ghrelins; and Fig. 6f, pancreatic polypeptide)^12–16^. As the purpose of this experiment was to examine whether the improved WB is experimentally effective, the use of 100 ng of protein content, which is an abundant amount for general WB detection, was fixed for all peptide hormones and analogs. Because our (Fig. 3a,b) and other investigators’ results^7–10^ indicated that the effect of aldehyde treatment on fixation has a preference, we confirmed the usability of the application under the fixation condition of 0.2% GA or 0.4% PFA. As shown in the results (Fig. 6), we could confirm signal enhancement by both or either of the aldehydes in the 11 peptide hormones and analogs (Fig. 6a–d,f) except for desacyl ghrelin (Fig. 6e). As summarized in Table 1, the results clearly demonstrate that there is a preference in the fixation step of peptides.

**Table 1:** Usability of the improved WB for detection of peptide hormones involved in diabetes.

Peptide hormones and analogs			0.2% GA	0.4% PFA
Insulin	Proinsulin^12^		+*	ND
	Insulin^12^		++	++
	Insulin analogs^13^	Insulin glulisine	++	++
		Insulin detemir	+	+
		Insulin degludec	+	+
Glucagon^12^			++	+
GLP-1	GLP-1 (1–36) amide^12, 14^		++	+
	GLP-1 (7–36) amide^12, 14^		++	+
Somatostatin	Somatostatin-28^12^		+	+
	Somatostatin-14^12^		+	+
Ghrelin	Ghrelin^15^		++	−
	Desacyl-ghrelin^15^		−	−
Pancreatic polypeptide^16^			++	−

These results were evaluated under a condition using 100 ng of each peptide sample. ++, more effective; +, effective; −, not or less effective; ND, not determined; *Results from Fig. 5a.

### Detection of diabetes-associated peptide hormones in cell lines, whole pancreas, and islets

**Figure 7:**
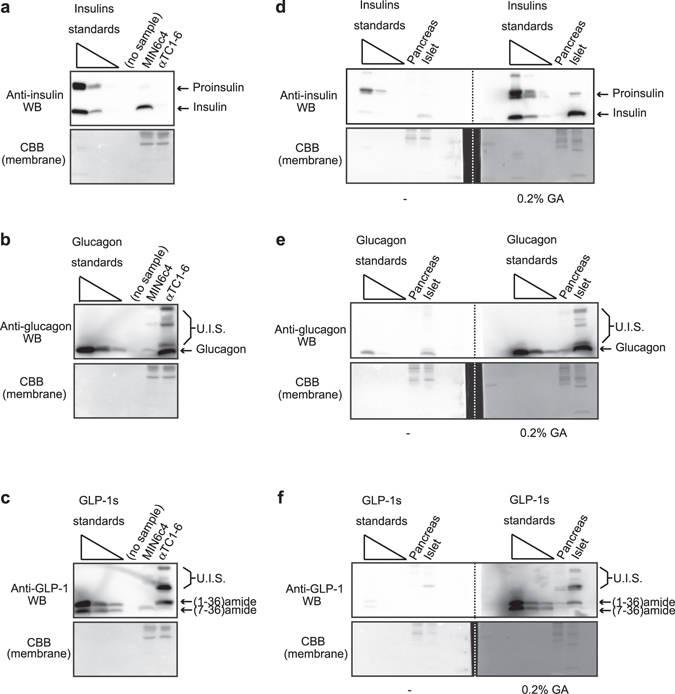
Application of the improved method for cell lines, pancreas, or islet samples shows versatility of the method. (**a**) Insulins, (**b**) glucagon, and (**c**) GLP-1 proteins in MIN6c4 and αTC1-6 cell lysates (5 μg) were detected using the improved WB (fixation, 0.2% GA). The loaded standards were as follows: proinsulin/insulin, 51.7 to 0.5 pmol (each 1/10 dilution, 3 points); glucagon, 287.1 to 2.9 pmol (each 1/10 dilution, 3 points); and GLP-1 (1-36) amide/GLP-1 (7-36) amide, 30.3 to 0.3 pmol (each 1/10 dilution, 3 points). CBB staining of the blotted membrane was performed to ensure appropriate loading. (**d**) Insulins, (**e**) glucagon, and (**f**) GLP-1s in whole mouse pancreas and islets were analyzed by WB with or without the additional steps. The loaded standards were as follows: proinsulin/insulin, 51.7 to 0.5 pmol (each 1/10 dilution, 3 points); glucagon, 287.1 to 2.9 pmol (each 1/10 dilution, 3 points); and GLP-1 (1-36) amide/GLP-1 (7-36) amide, 91.0 to 0.9 pmol (each 1/10 dilution, 3 points). CBB staining of the blotted membrane was performed to ensure appropriate loading. The WB images in each sub-part of the figure were captured at one time. U.I.S., unidentified signals.

Finally, we evaluated whether the improved WB is experimentally suitable for the detection of *in vitro* or *in vivo* sample datasets other than insulins in MIN6c4 cells. In diabetes studies, MIN6 lines from a β cell origin^17^ and αTC1 lines from an α cell origin^18^ have been well characterized as cell lines established from mice pancreatic islets. For this experiment, we selected MIN6c4 and αTC1–6 sub-cell lines, which have been cloned from those parental lines^11, 19^, and examined protein levels of insulins (proinsulin and insulin), glucagon, and GLP-1s (GLP-1 [1–36] amide and GLP-1 [7–36] amide) (Fig. 7a–c). Insulins protein expression appeared to be exclusive in MIN6c4 cells (Fig. 7a). Although negligible glucagon protein expression (no more than 1/100 compared with that in αTC1-6 cells) was detected in MIN6c4 cells, the expression was almost exclusive in αTC1-6 cells (Fig. 7b). Additionally, in αTC1-6 cells, we found some chemiluminescent signals from anti-glucagon reactive protein whose molecular weights are higher than that of mature glucagon (shown as unidentified signals in Fig. 7b). We also examined GLP-1s expression because previous studies have indicated GLP-1 expression in not only intestinal L cells but also pancreatic α cells^20–22^ (Fig. 7c). Although the apparent molecular weight of the strongest chemiluminescent signal in αTC1-6 cells was larger than that of either GLP-1 (1-36) amide or GLP-1 (7-36) amide (shown as one of unidentified signals in Fig. 7b), a protein band of which mobility corresponds to that of GLP-1 (1-36) amide was clearly detected in αTC1-6 cells (Fig. 7c). Also, we managed to identify a protein band that seems to be from GLP-1 (7-36) amide in not only αTC1-6 cells but also MIN6c4 cells, whereas that in both cells was very low. Next, we applied the improved WB to the detection of diabetes-associated proteins in mice pancreas or islet lysate (Fig. 7d–f). Importantly, the signal patterns of WB using islet lysates as a protein sample correlated significantly with those using cell line lysates as a protein sample as shown in Fig. 7a–c: anti-insulin WB of islets and MIN6c4, anti-glucagon WB of islets and αTC1-6, and anti-GLP-1 WB of islets and αTC1-6 cells. Hence, we regard the unidentified signals as coming from precursors or intermediates of the peptide hormones. When performing WB of islets lysate with anti-somatostatin, we detected an extremely low level of a reactive signal of which mobility in gel electrophoresis corresponded to that of somatostatin-14 (Supplemental Fig. 5). Considering that this result is highly reproducible and somatostatin-14 is a major form of somatostatin in islets^12^, we now regard the signal as probably coming from endogenous somatostatin-14. Unfortunately, we could not obtain any signals from WB of islets with anti-ghrelins and anti-PP antibody (data not shown). These results show the usability of the improved WB for the detection of other diabetes-associated peptide hormones in various biological samples.

## Discussion

We demonstrated that the WB sensitivity of insulin and the other peptide hormones involved in diabetes was improved by the addition of extra steps to the general WB procedure. Sample preparation in the improved method is essentially identical to that of the Laemmli method, which is used in a general SDS-PAGE. In the quantification of proinsulin/insulin by ELISA, sample preparation by acid/ethanol extraction has been recommended because the partial purification aims to prevent the insulin-bound proteins from affecting the antigen-antibody reaction and the other proteins from causing non-specific antigen-antibody reactions. In particular, it has been reported that insulin tends to form fibrils (or aggregation) under a certain condition^23^. Furthermore, such sample pretreatment often tends to perturb the quantitative performance. The fact that an extra procedure is not required for sample preparation in the modified WB means that the prepared samples can be used for WB of the other proteins. Additionally, in the case in which the difference in mobility in the electrophoresis among forms of a hormone member is enough to cause separation of the WB signals (proinsulin/insulin, Fig. 5a: GLP-1 (1-36) amide/GLP-1 (7-36) amide, Fig. 6c: somatostatin-28/somatostain-14, Fig. 6d: ghrelin/desacyl ghrelin, Fig. 6e), if we can use an antibody that recognizes the targeted forms of a hormone member, these proteins can be distinguished and analyzed in one sample lane. Importantly, the effect of aldehyde on the signal enhancement showed diversity as described below (Table 1). Insulin and its analog WB are improved to a similar extent by both GA and PFA fixation, meaning that there is little preference as to the types of aldehyde used in insulin WB. Interestingly, in the case without the “pre-blocking step,” PFA fixation was not effective in improving the insulin WB (Fig. 3a,b). This observation shows that “scaffolds for fixation” supplied by the pre-blocking step may contribute to relieving the preference as to the types of aldehyde. Although ghrelin and desacyl ghrelin have the same amino acid sequence except for acylation of the serine residue at position 3, only ghrelin WB but not desacyl ghrelin was enhanced by 0.2% GA fixation. Considering that the ghrelin intensity approached the desacyl ghrelin intensity by GA fixation, we wonder whether desacyl ghrelin is retained on the blotting membrane without the fixation step. This result may mean that acylation of a protein has an inhibitory effect on the retention of a protein to the blotting membrane.

The method was applicable to various biological samples such as cell line lysates (Figs 5g and 7a–c), cell culture supernatants (Fig. 5h), whole pancreas lysates, and islet lysates (Fig. 7d–f). Unfortunately, we failed to detect insulins in mice serum with experimental equipment and reagents that we can prepare now (Supplemental Fig. 3). In the present study, the carry-in volume of mice serum as a sample was restricted to 1 μL. Considering the SDS concentration in the used sample buffer (1%), the general protein concentration in mice serum (60 µg/µL), and the binding ratio of SDS to protein (1.4 g SDS/g protein), the use of 1 μL serum in a 10-μL applied volume (a mini-gel scale) is almost at the allowable limit (theoretical limit, 71.4 μg protein/10 μL sample buffer). Supplemental Fig. 3 also shows that the detection limit of proinsulin and insulin with experimental equipment and reagents that we possess now is approximately 15 fmol (=approx. 30 pg)/lane. For example, Hoffler *et al*. reported that serum insulin levels of C57BL/6 fed a normal-fat or high-fat diet are 10–25 amol/μL (=approx. 20–50 fg/μL) or 15–100 amol/μL (=approx. 30–200 fg/μL), respectively^24^. Considering that 1 μL of serum is the allowable limit, a sensitivity of 10–100 amol (=approx. 20–200 fg)/lane would be required for the detection of signals. Hence, we have to improve the sensitivity by a factor of at least 10-fold. To take maximum advantage of the improved WB, which is not required for additional procedures for sample preparation, sensitization of chemiluminescent detection system such as equipment or reagents is likely to be more meaningful than the concentration of protein solution by pretreatment. Nevertheless, when serum samples were concentrated to an appropriate level by appropriate procedures, we would be able to apply our technique to qualitative analyses such as detection of abnormal insulin processing. Importantly, considering the allowable limit of a protein concentration in sample preparation of SDS-PAGE, albumin elimination from serum samples would be essential.

As shown in Fig. 7, we were likely to detect various specific signals (shown as unidentified signals) in WB of both cell lines and tissue lysates. For example, if we subject the specimens to two-dimensional SDS-PAGE, we may be able to identify unknown precursor, intermediate, or modified forms of the peptide hormones.

The modified WB can contribute to qualitative or quantitative analyses of diabetes-associated peptides by providing analytical information based on electrophoresis. Data supplied by ELISA is merely numerical, which is the weakest point of ELISA. The fact means that the quantification of hormones should be confirmed not only by ELISA but also by other experimental methods as our method. The improved WB is valuable in providing another method in the immunological assay of diabetes-associated peptide hormones other than ELISA and may contribute to precise interpretations of the results obtained by the immunological experimental methods in the area of diabetes research.

## Methods

### Peptide hormones

Organism species of all peptide hormones used in the present study were human: proinsulin (AmideBio), insulin (Wako), glucagon (Peptide Institute), GLP1 (1–36) amide and GLP1 (7-36) amide (KareBay Biochem), somatostatin-28 and somatostatin-14 (KareBay Biochem), desacyl ghrelin and ghrelin (Peptide Institute), and pancreatic polypeptide (KareBay Biochem). Insulin detemir (Novo Nordisk), insulin glulisine (Sanofi), and insulin degludec (Novo Nordisk), which are used clinically, were used as representative insulin analogs.

### Antibodies

Primary antibodies used in this study were as follows: anti-insulin rabbit mAb clone c27c9 (Cell Signaling Technology), anti-glucagon rabbit mAb clone EP3070 and anti-GLP1 mouse mAb clone 4F3 (Abcam), anti-ghrelin mouse mAb clone 1ML-1D7 (Millipore, USA), anti-pancreatic polypeptide goat pAb (R&D Systems), and anti-somatostatin 14 rabbit pAb (Bioss).

### Preparation of peptide hormone standards

The proteins were mixed with SDS sample buffer (75 mM Tris-HCl at pH 6.8, 1% SDS, 10% glycerol, 2.5% sucrose) containing 1 µg/mL BSA as a protein carrier unless described otherwise. After the addition of 2-mercaptoethanol (final, 5%) and CBB G-250 (final, 0.00125%), the proteins were boiled for 5 min.

### Preparation of cell, pancreas, and islet lysates

MIN6c4 and αTC1-6 cells were maintained with Dulbecco modified Eagle’s medium containing 15% fetal bovine serum, 12.5 mM glucose, 1 mM sodium pyruvate, 1% penicillin, and 100 μM 2-mercaptoethanol. Mouse islets were obtained from C57BL/6 mice (3 to 12 months old, both sexes) by a collagenase/hand-picking method reported previously^25^. The animal experiment was performed in accordance with the provisions of the Ethics Review Committee for Animal Experimentation at the Tokyo University of Science.

The cell or tissue pellets were lysed by the addition of SDS sample buffer, boiled for 5 min, and sonicated. Protein concentrations were determined by BCA protein assay (Thermo Fisher Scientific) according to the manufacturer’s protocol and were adjusted by the addition of lysis buffer. 2-mercaptoethanol (final, 5%) and CBB G-250 (final, 0.00125%) were subsequently added to the protein solutions, and the mixtures were boiled for a further 5 min. Five micrograms of protein was subjected to WB.

### Preparation of cell culture supernatants

For the cell culture supernatant, a glucose-stimulated insulin secretion assay was performed. MIN6c4 cells were seeded into a 12-well plate at 5.8 × 10^5^ cells per well. On the next day, the culture medium was changed to 1 mL of Krebs-Ringer bicarbonate HEPES (KRBH) buffer (1.19 mM KH_2_PO_4_, 25 mM NaHCO_3_, 119 mM NaCl, 4.74 mM KCl, 1.19 mM MgCl_2_, 2.54 mM CaCl_2_, 10 mM HEPES at pH7.4, 0.1% BSA) containing 5 mM glucose and incubated for 45 min in a CO_2_ incubator. After the preincubation, the cells were cultured in 1 mL of KRBH buffer containing 5, 12.5, or 30 mM glucose for a further 2 h in a CO_2_ incubator. Then, the cell culture supernatants were collected as samples. The cultured cells were harvested, and the total proteins per well were determined for loading normalization of the cell culture supernatant samples. For an analysis, a cell culture supernatant corresponding to 2 μg of the total protein was used.

### Tris/Tricine/urea SDS-PAGE

The separation of proteins was performed in a 10–20% gradient polyacrylamide gel containing 6.3 M urea with the Tris/Tricine/SDS buffer systems. The gradient gel was made using the Gel Factory mini MP system (DRC). Electrophoresis was performed using an XV PANTERA MP SYSTEM (DRC). Materials other than the running buffer (50 mM Tris-HCl at pH 8.3, 50 mM Tricine, 0.5% SDS, 5 mM NaHSO_3_) were prepared as described elsewhere^4^.

### Western blotting

The gels after electrophoresis were blotted to PVDF membranes using a wet-type blotting system, Criterion Blotter with Plate Electrodes (Bio-Rad). The transfer buffer formulation used was as follows: 25 mM Tris-HCl at pH 8.3, 192 mM glycine, 0.05% SDS, 20% methanol. In general, in the WB procedure, the blotting step is followed by the blocking step. In the improved WB procedure for proinsulin/insulin and the other diabetes-associated peptide hormones, extra experimental procedures were added between the blotting and the blocking step as follows. The blotted membrane was briefly washed in TTBS (TBS [50 mM Tris-HCl at pH 7.4, 150 mM NaCl] containing 0.1% Tween 20) and soaked with blocking buffer (1% skim milk and 0.1% BSA in TTBS) for 5 min. After a 3-min washing step with TPBS (PBS [10 mM Na_2_HPO_4_, 1.8 mM KH_2_PO_4_, 137 mM NaCl, 2.7 mM KCl at pH 7.4] containing 0.1% Tween 20), the membrane was incubated in 0.2% GA or 0.4% PFA in TPBS for 15 min. The aldehyde-treated membrane was briefly washed 3 times with TPBS and then immersed in citrate retrieval buffer (10 mM citric acid at pH 6.0, 1 mM EDTA, 0.05% Tween 20). Retrieval was performed using a microwave oven (for 10 min at 600 W after boiling). After the retrieval solution cooled to room temperature, the membrane was soaked with quenching buffer (200 mM glycine in TPBS) for 10 min (the additional steps in the improved WB are until this step). The membrane was incubated in blocking buffer for 30 min and probed with the appropriate primary antibody overnight at 4 °C. After washing 3 times with TTBS, the membrane was incubated with the appropriate secondary antibody, horseradish peroxidase-conjugated F(ab’)_2_ fragment of goat anti-mouse IgG, anti-rabbit IgG, or anti-goat IgG (Jackson ImmunoResearch Laboratories), for 1 h at room temperature. After washing with TTBS, the membranes were incubated with ImmunoStar Zeta reagent (WAKO). The specific proteins were visualized with Ez-Capture MG (ATTO).

### Quantification

The intensity of specific bands was measured using CS analyzer software (ATTO). A calibration curve was obtained by four-parameter logistic fitting methods^26^, and parameters of the equation were calculated by using the Solver add-in of Excel for Mac 2011 (Microsoft).

## Electronic supplementary material

Supplemental Figures
