# Supplementary material for: Modified Western blotting for insulin and other diabetes-associated peptide hormones
Source: Sci Rep. 2017 Jul 31;7:6949. doi: 10.1038/s41598-017-04456-4 (PMC5537366; doi:10.1038/s41598-017-04456-4)

Supplemental Figures

Title

Modified Western blotting for insulin and other diabetes-associated peptide hormones

Authors

Naoyuki Okita, Yoshikazu Higami, Fumio Fukai, Masaki Kobayashi, Miku Mitarai, Takao Sekiya, Takashi Sasaki

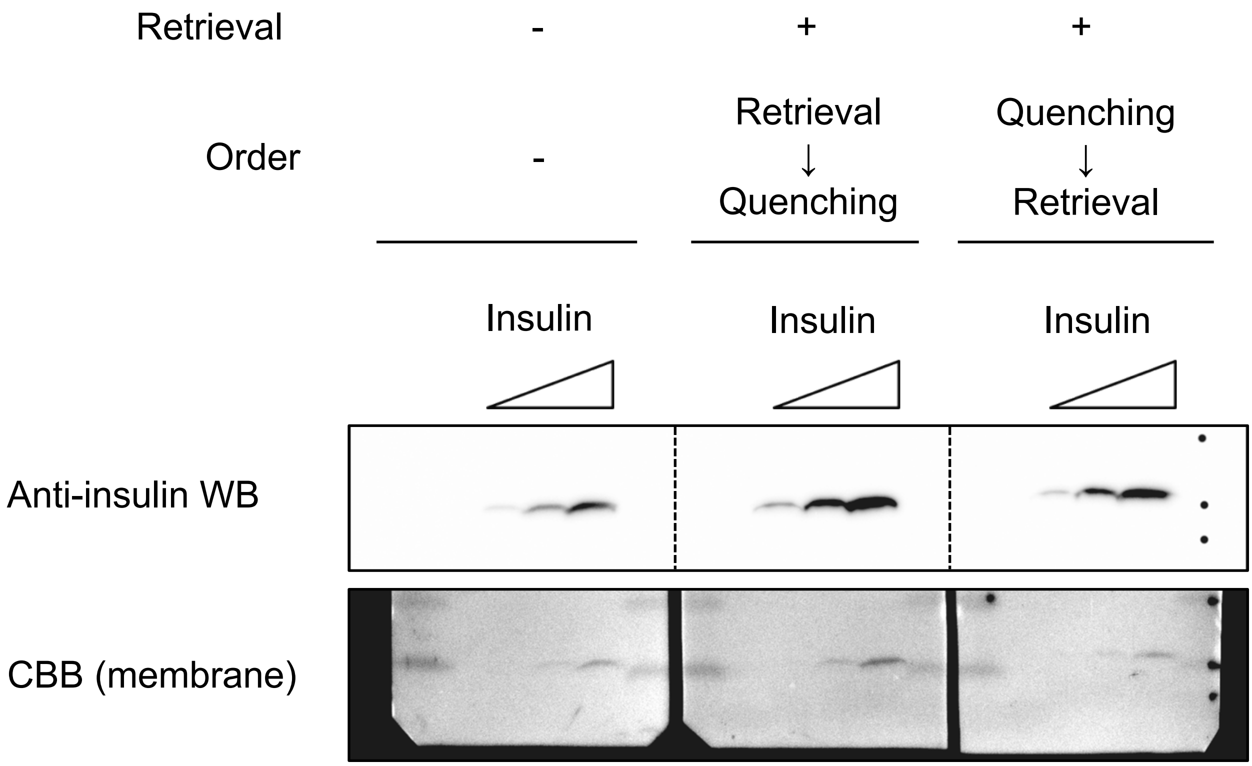

Supplemental Figure 1. Glycine quenching is effective when performed following the retrieval step. Dilution series (30, 100, 300 ng) of insulin were subjected to Tris/Tricine/urea SDS-PAGE. After electro-blotting/GA fixation, the one blotted membrane was separated into 3 slips for the control experiment (not subjected to retrieval and quenching steps) and the described experiments. To compare the effects of various conditions, WB images in each sub-part of the figure were captured at one time.

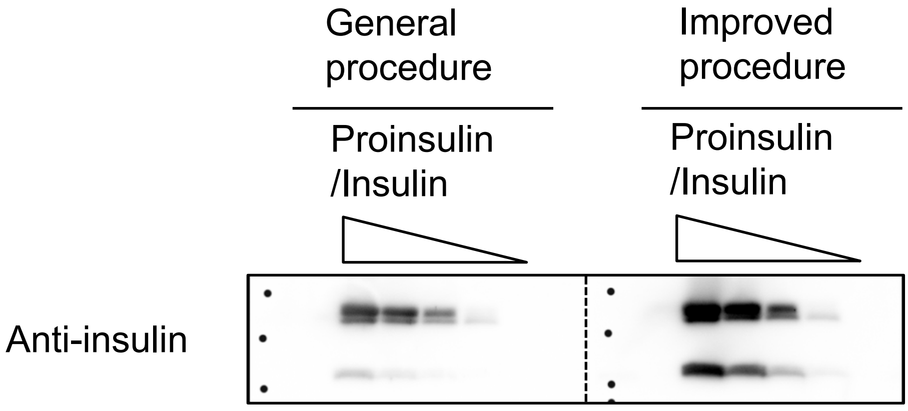

Supplemental Figure 2. The improved methods are also effective when using another antibody against insulin (clone L6B10). Dilution series of equal moles of proinsulin and insulin (range, 51.1 to 0.5 pmol) were subjected to the general or the improved WB procedure with anti-insulin clone L6B10. The blotted slips for the general or improved protocol were separated from one blotted membrane.

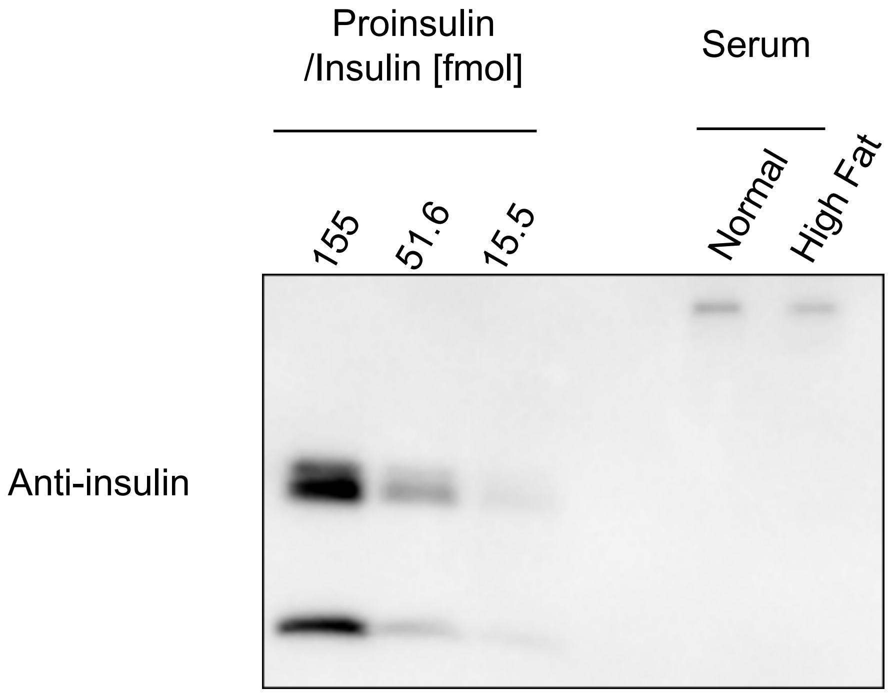

Supplemental Figure 3. Application of the improved method for mouse serum sample. (a) Proinsulin/insulin standards were used at a range of 155-15.5 fmol (Proinsulin, 485 -48.5 pg; Insulin, 300-30 pg). As a serum sample, 1 μL of pooled serum (n=9) from normal diet-fed or high fat diet-fed C57BL/6 mice (both sexes, 22 weeks old) was prepared. Blood sampling was performed under no regulation of food intake. The samples were subjected to the improved WB.

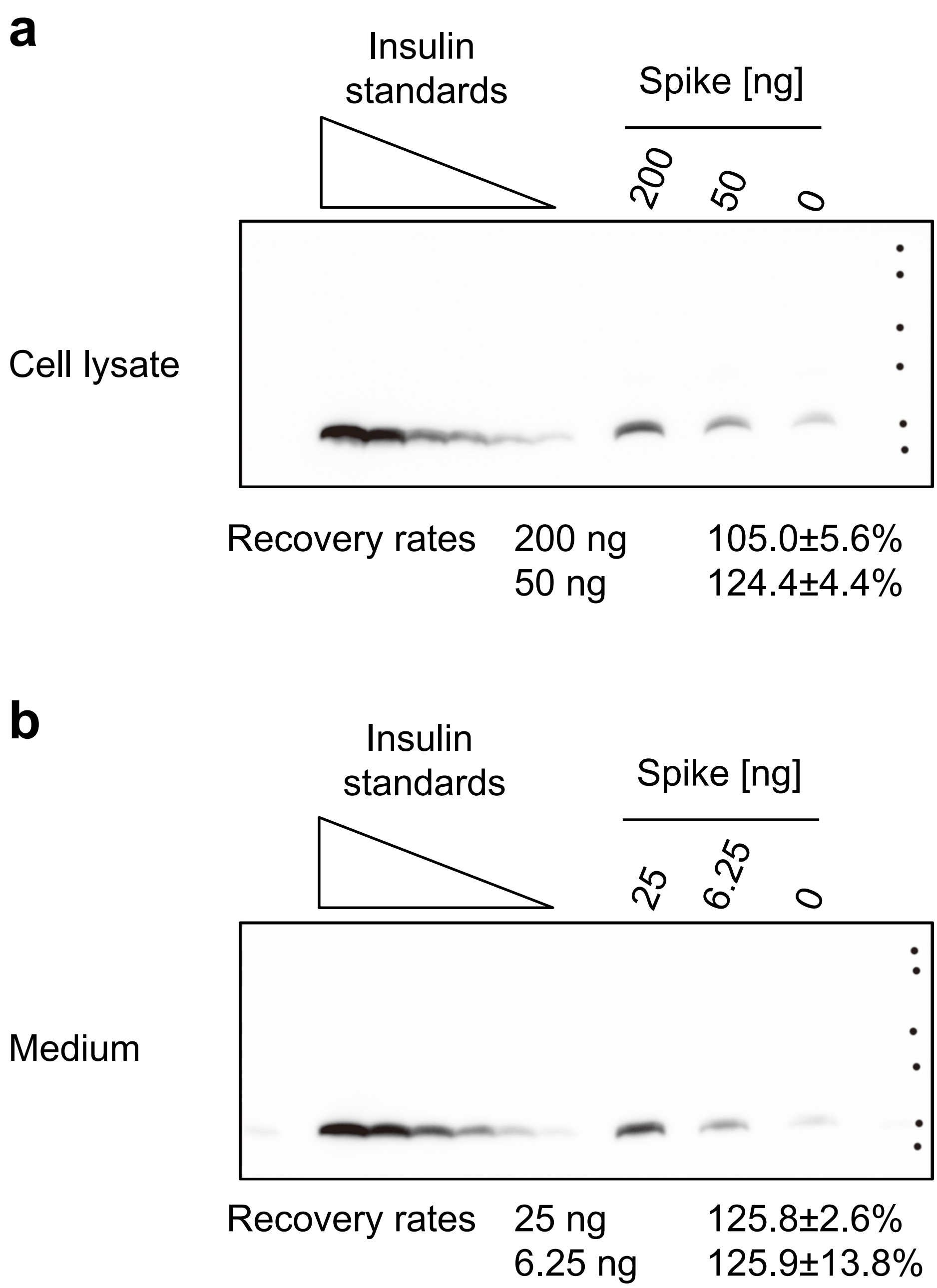

Supplemental Figure 4. Spike and recovery experiment in the improved method. Spike and recovery experiments were performed using MIN6c4 cell lysate (a) or culture medium (b). (a) Insulin standards were used at a range of 1000-31.25 ng (each 1/2 dilution, 6 points). As a cell lysate sample, 5 µg of extracted protein of MIN6c4 cells maintained with DMEM containing 12.5 mM glucose was prepared. Insulin spiking doses to cell lysates were 200 or 50 ng. (b) Insulin standards were used at a range of 125.0-3.90625 ng (each 1/2 dilution, 6 points). As a medium sample, a culture supernatant from MIN6c4 cells incubated with KRBH buffer containing 30 mM glucose for 2 h as shown in Methods was prepared. Insulin spiking doses to medium were 25 or 6.25 ng. The shown recovery rates (±standard error) were represented by averages from 4 independent data. The representative images were shown.

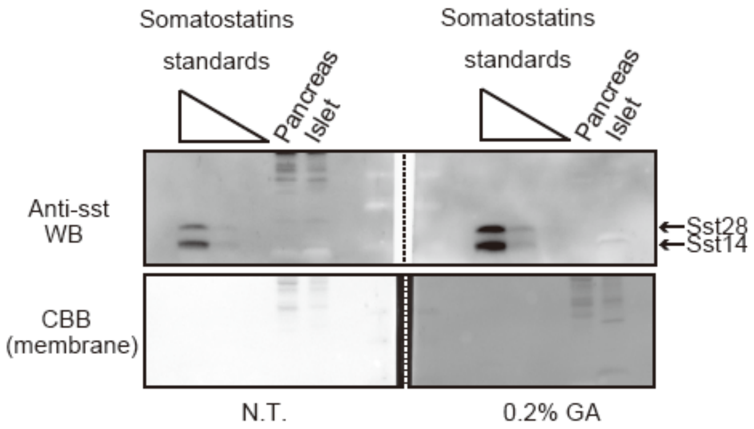

Supplemental Figure 5. WB of somatostatins in pancreas and islet sample. Somatostatins in mice pancreas and islet lysate (5 µg) were analyzed by WB with or without the additional steps. WB images in each sub-part of the figure were captured at one time. U.I.S. means unidentified signals. CBB staining of the blotted membrane were performed to ensure appropriate loading. The loaded standards of somatostatins were as follows: somatostatin-28/somatostatin-14, 611 to 6.11 fmol (each 1/10 dilution, 3 points).

Supplemental Information: Full Blot Images of WB in the Manuscript

Title
Modified Western blotting for insulin and other diabetes-associated peptide hormones

Authors
Naoyuki Okita, Yoshikazu Higami, Fumio Fukai, Masaki Kobayashi, Miku Mitarai, Takao Sekiya, Takashi Sasaki

Description
In the supplemental information, the original images (monochrome inversion) are shown without “auto-contrast adjustment” of Adobe photoshop software.

Seven-digit numbers described after figure numbers, the data acquisition date (YYYYMMDD):
Squares with bold solid lines, the location of images used in the main figures:
Dot lines, the location of borderline between the different membranes captured at one time:
Digital 6 dots traced in the WB images, the location of the colored molecular weight markers (42, 30, 16, 10, 4.6, and 1.7 kDa) (CST Cat. No.13070).

Fig.1 (20150217)

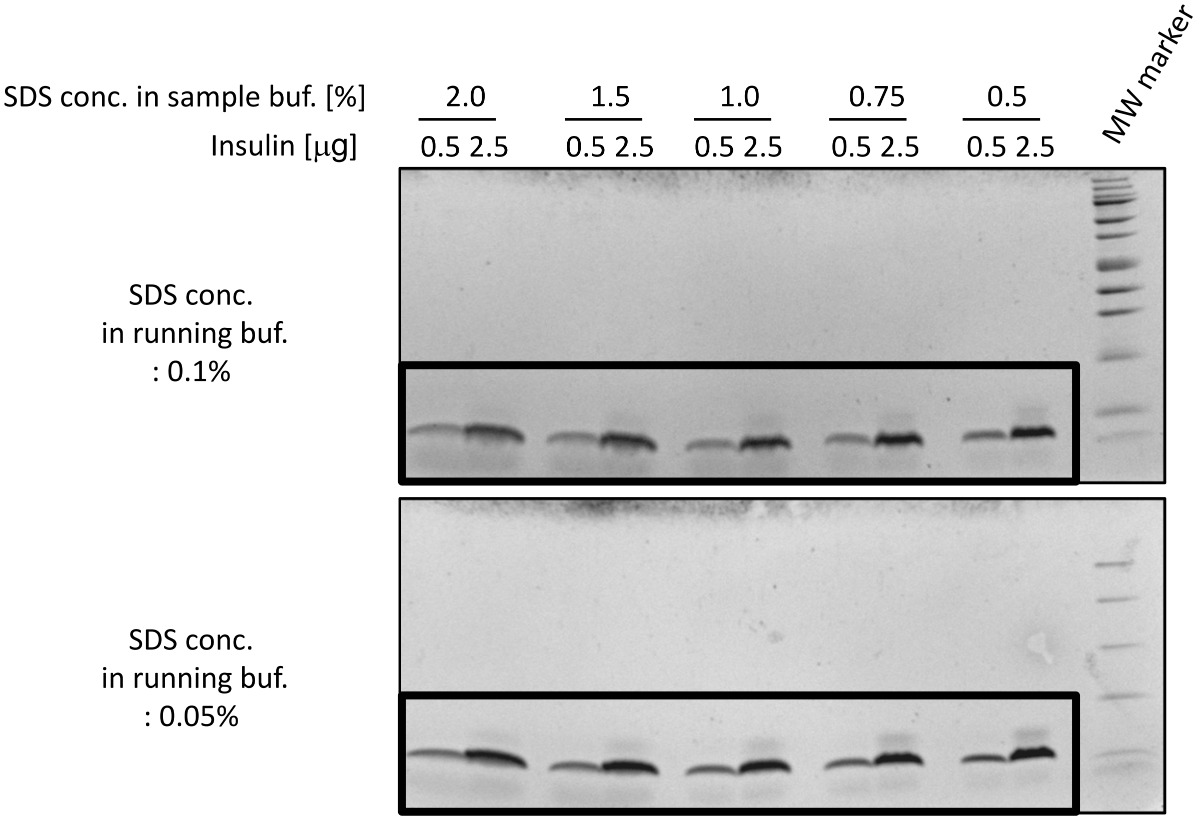

Fig.2a (20150328-1)

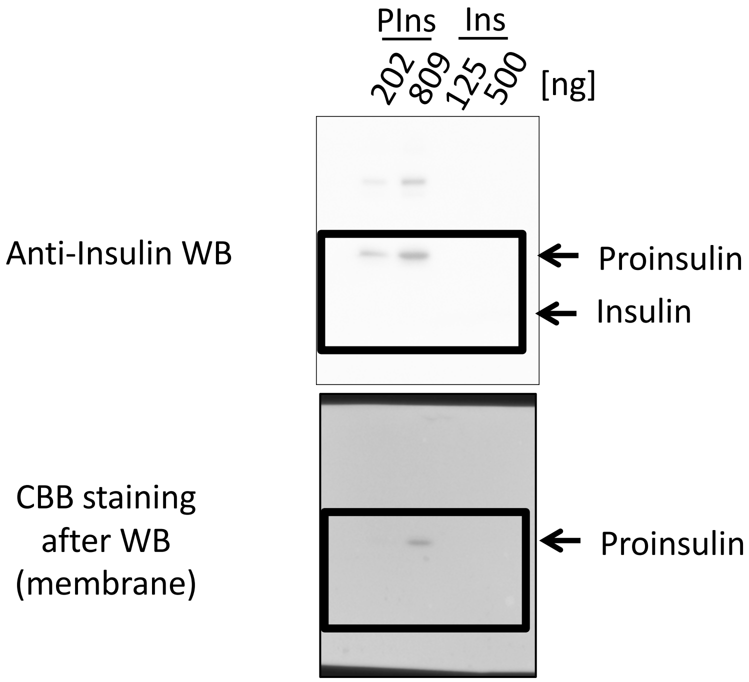

Fig.2b (20150327)

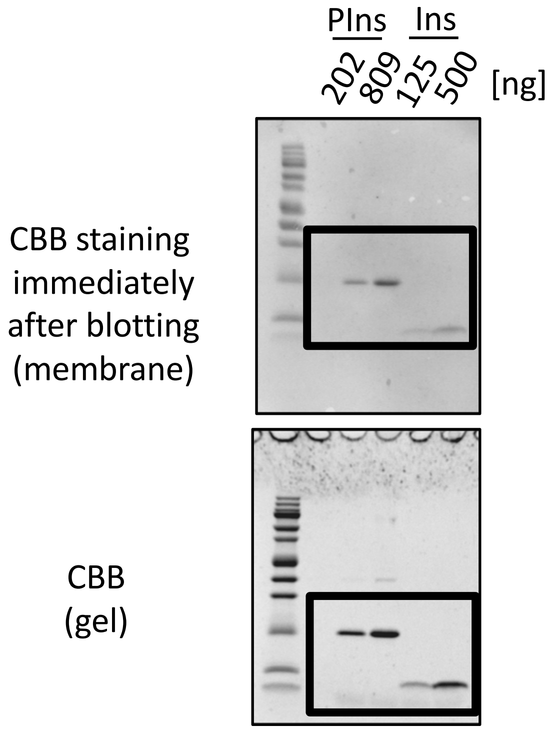

Fig.3a (20150319)

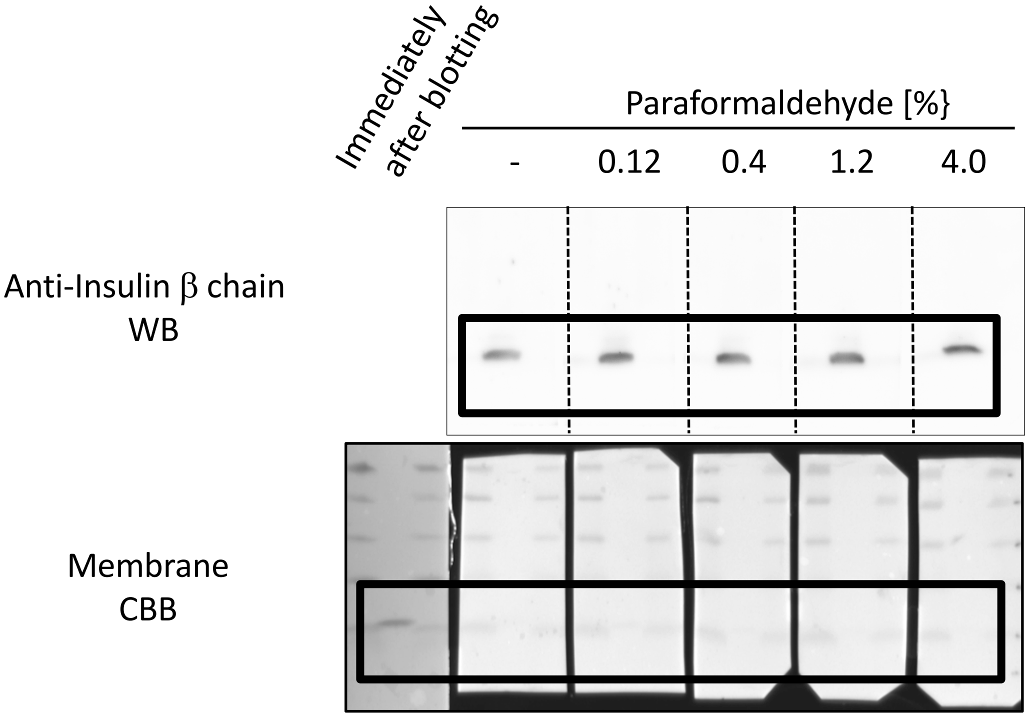

Fig.3b (20150319)

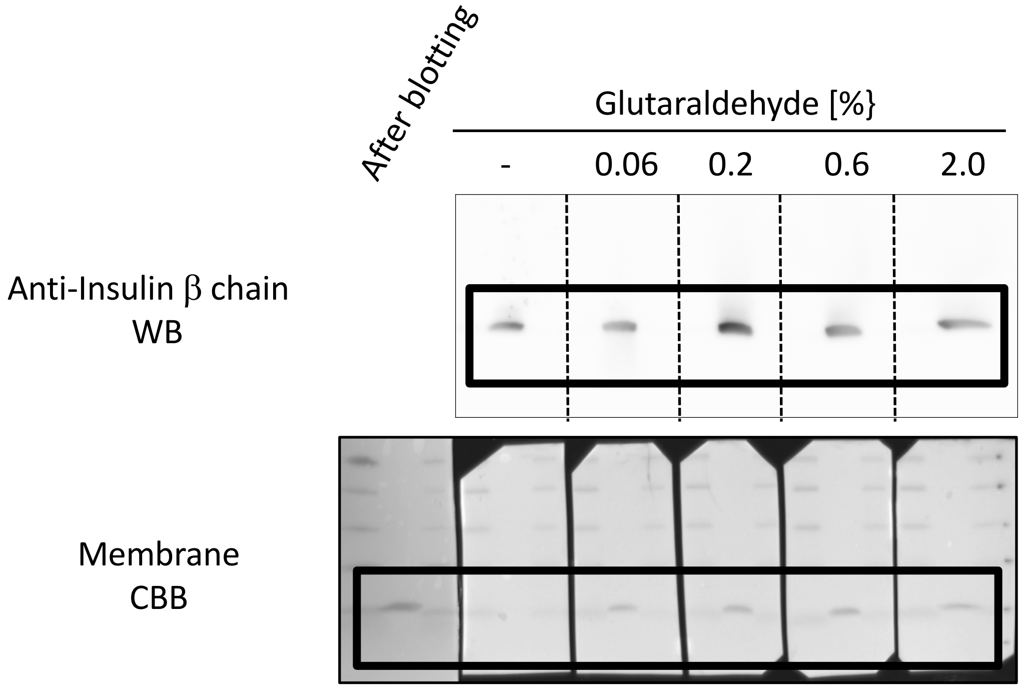

Fig.3c (20150422)

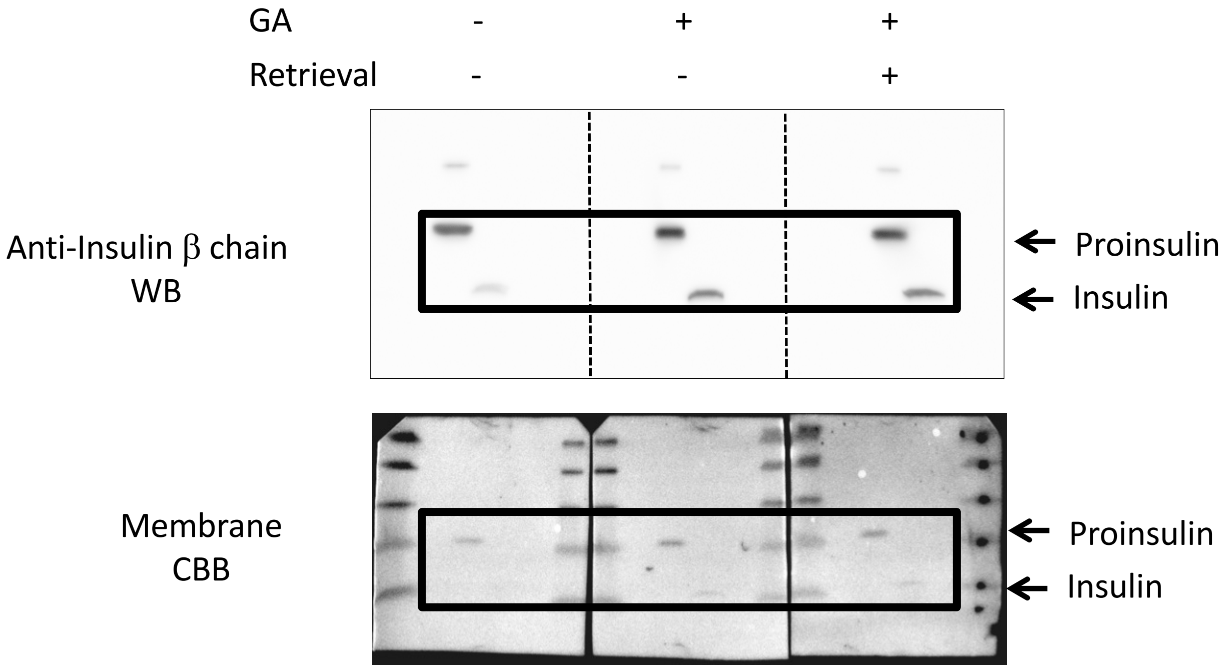

Fig.4a, b (20150717)

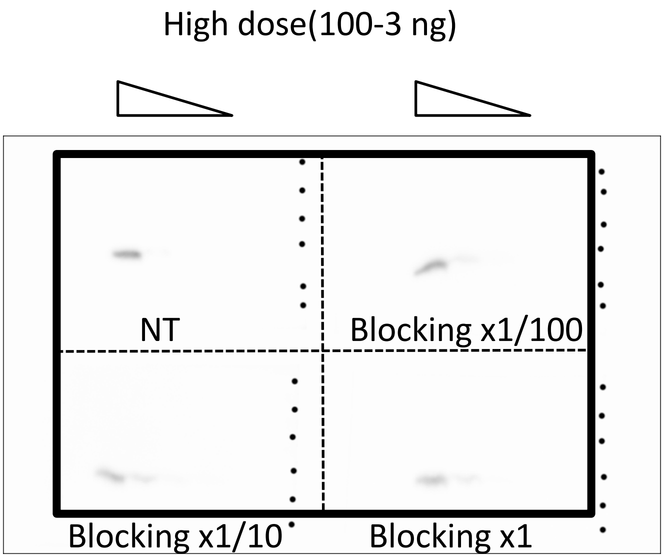

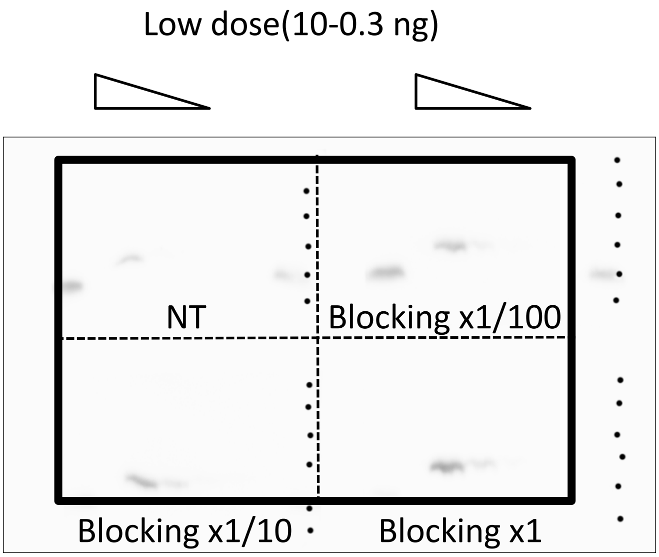

Fig.4c (20150826)

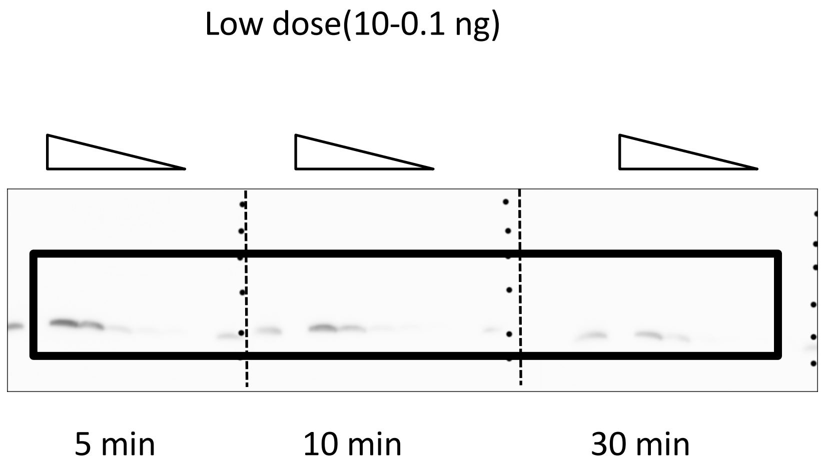

Fig.5a (20160414)

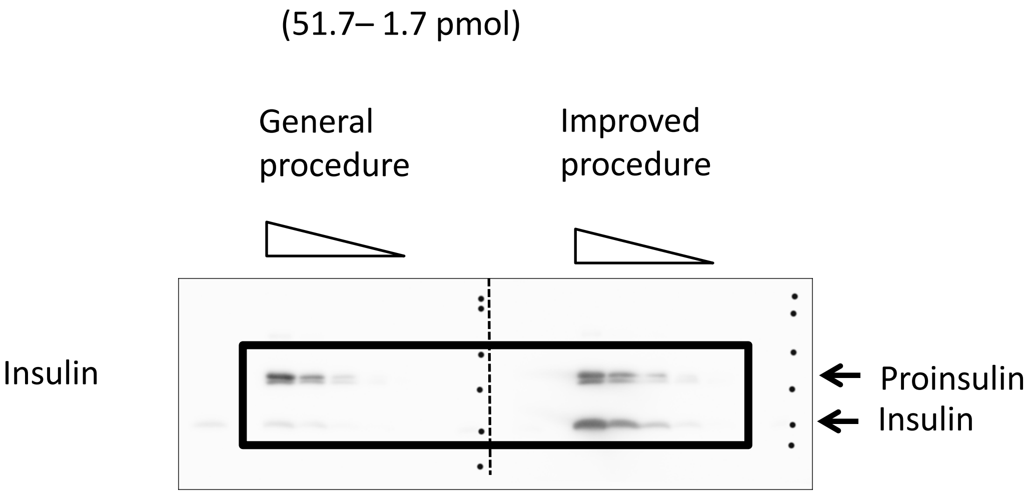

Fig.5g (20160422)

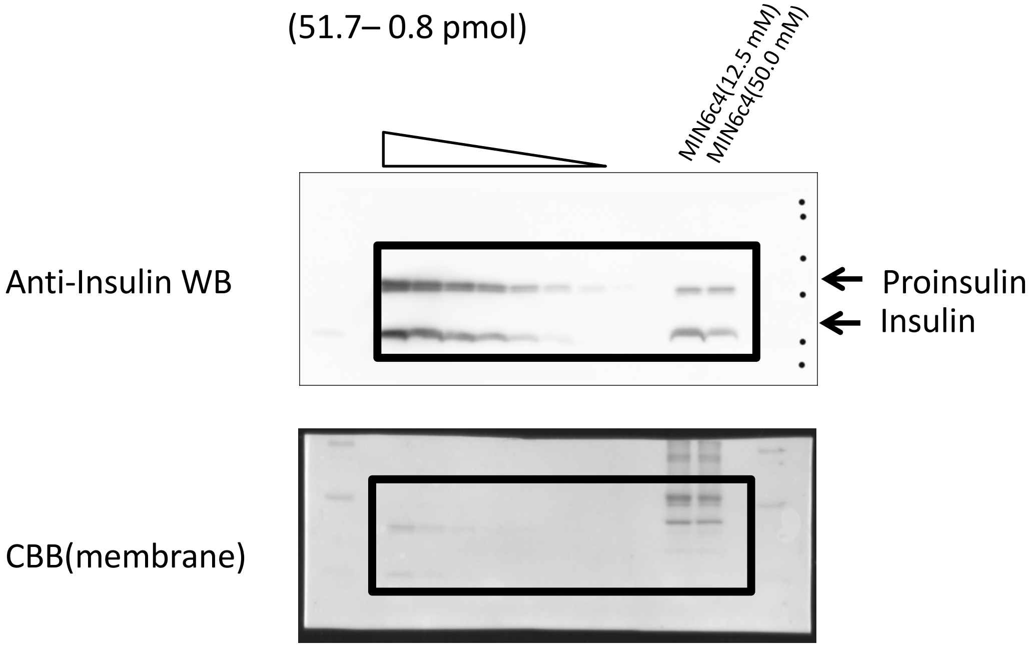

Fig.5h (20161123)

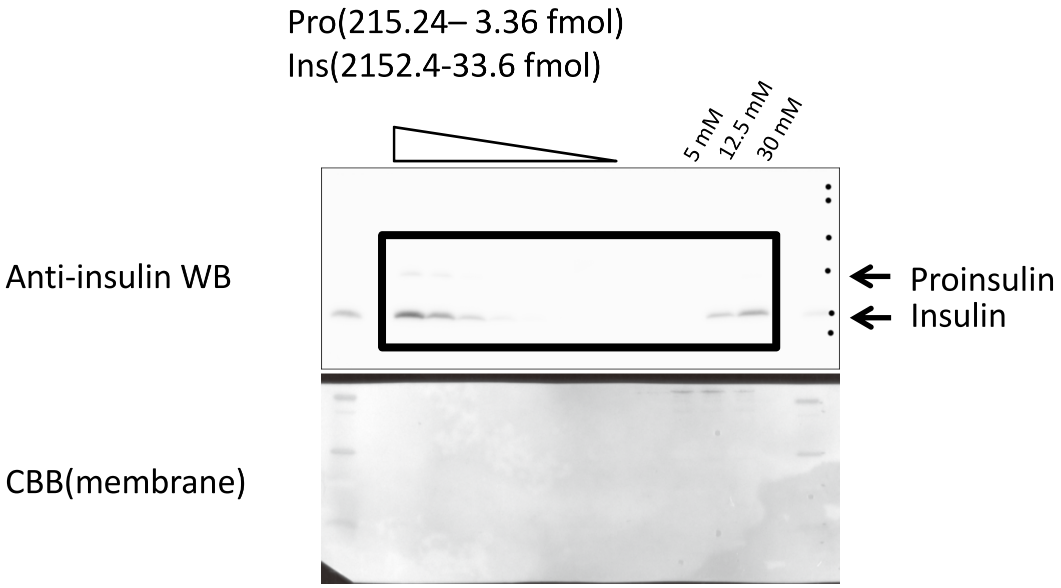

Fig.6a (20160326)

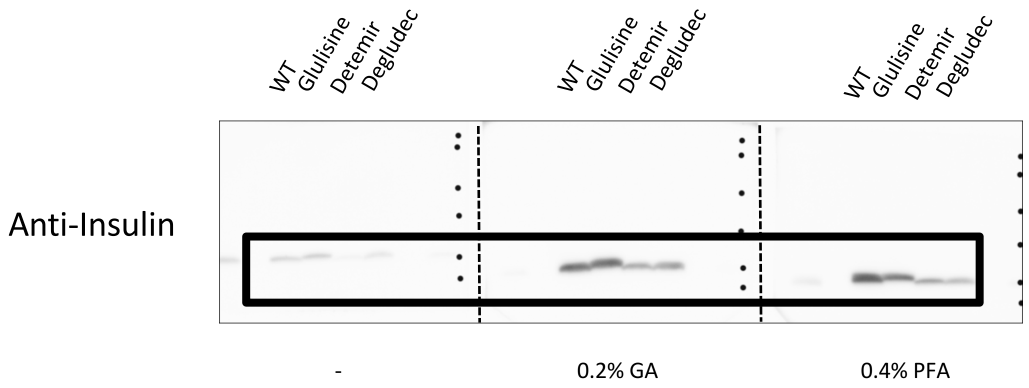

Fig.6b (20160326)

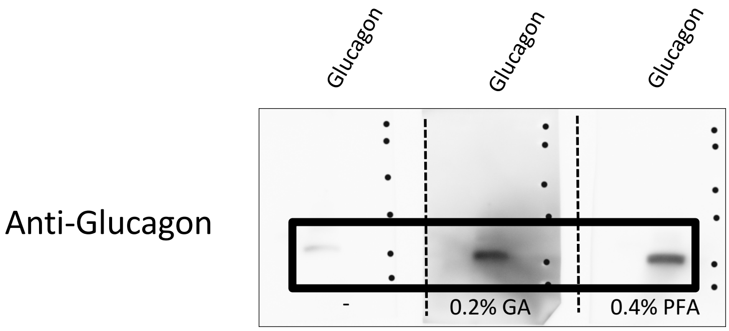

Fig.6c (20160326)

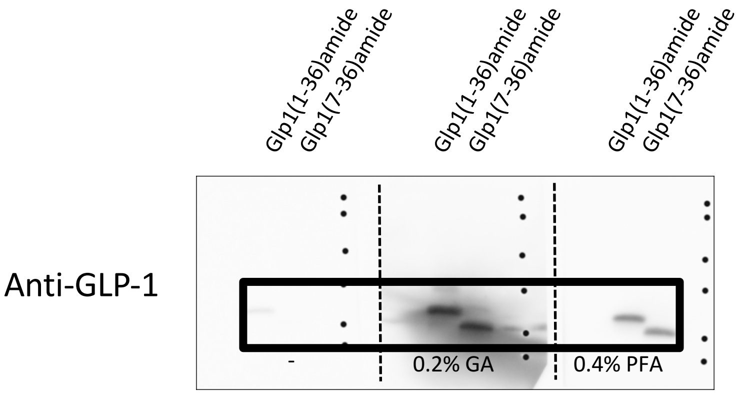

Fig.6d (20170118)

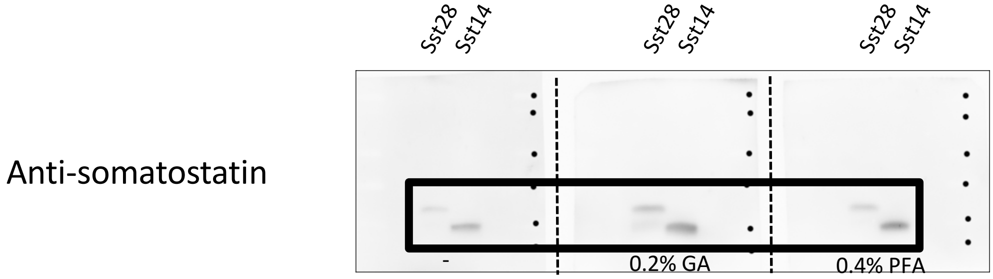

Fig.6e (20160330)

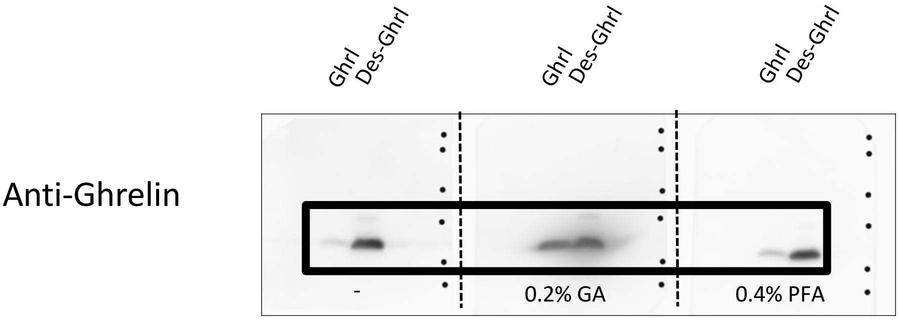

Fig.6f (20160330)

Anti-Pancreatic polypeptide

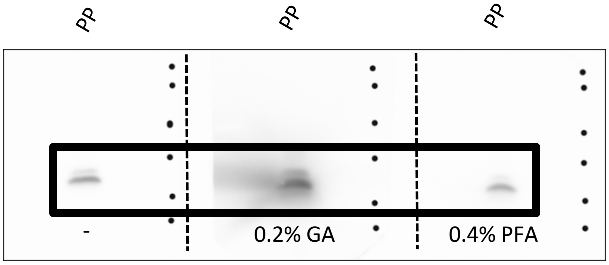

Fig.7a (20170107)

51.7-5.2 pmol

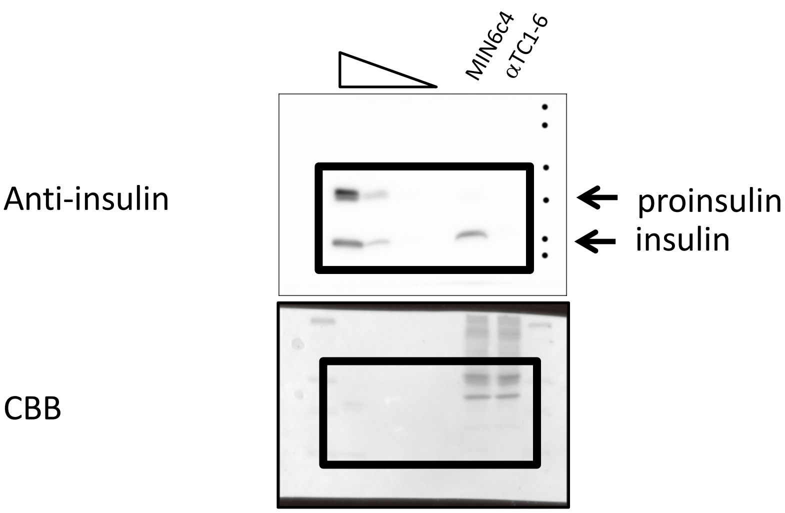

Fig.7b (20170107)

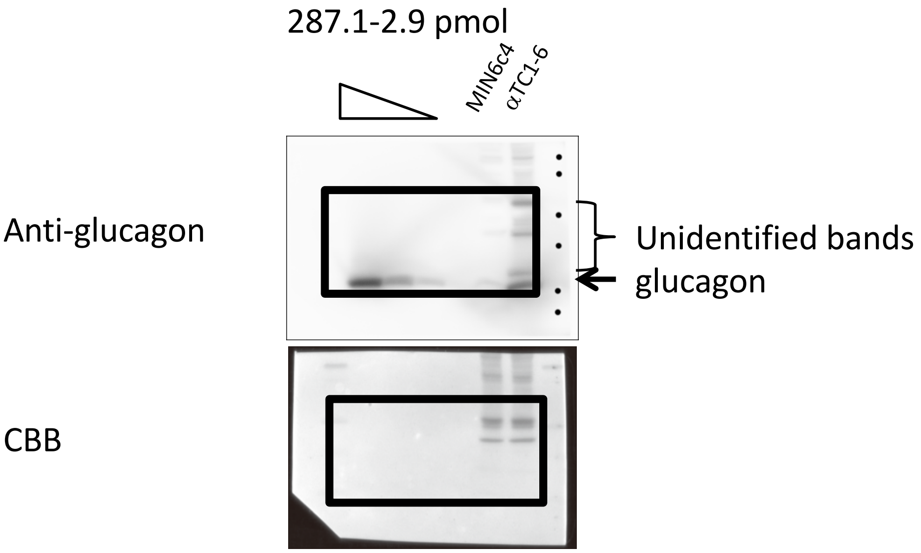

Fig.7c (20170107)

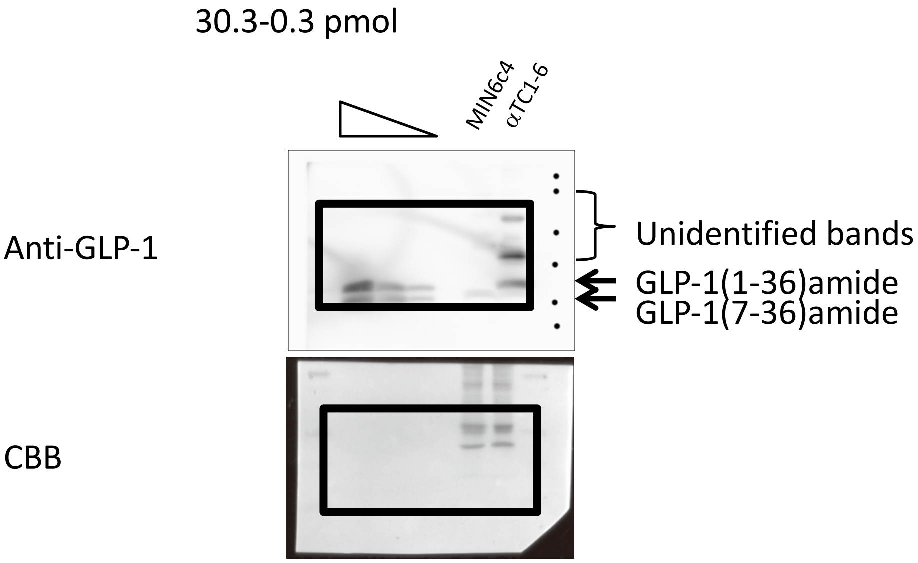

Fig.7d (20161201)

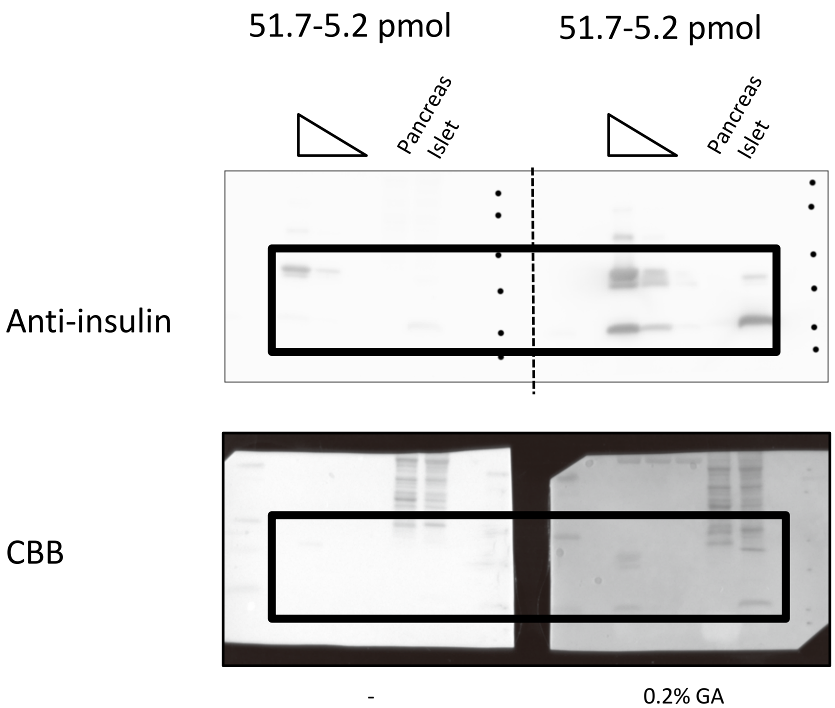

Fig.7e (20161201)

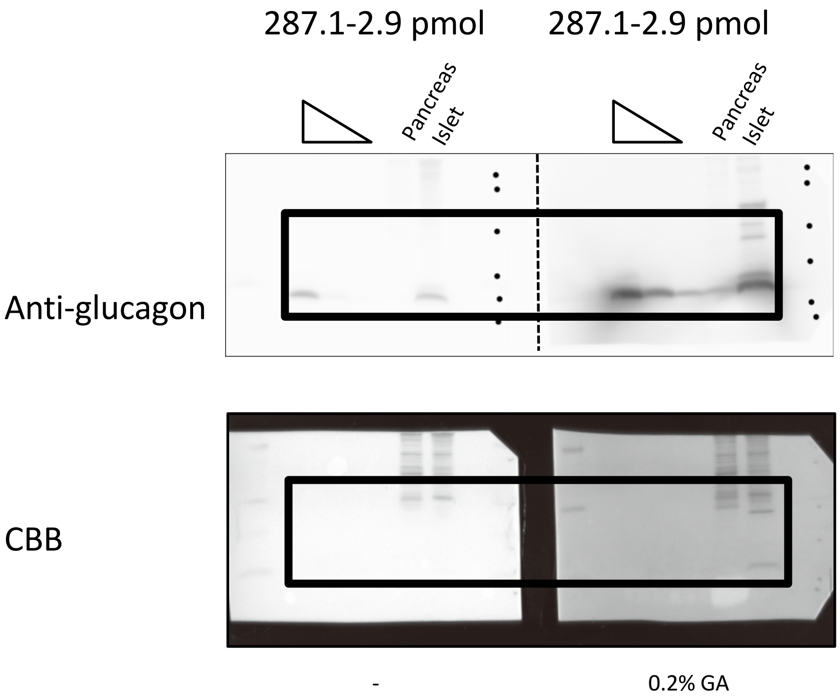

Fig.7f (20170118)

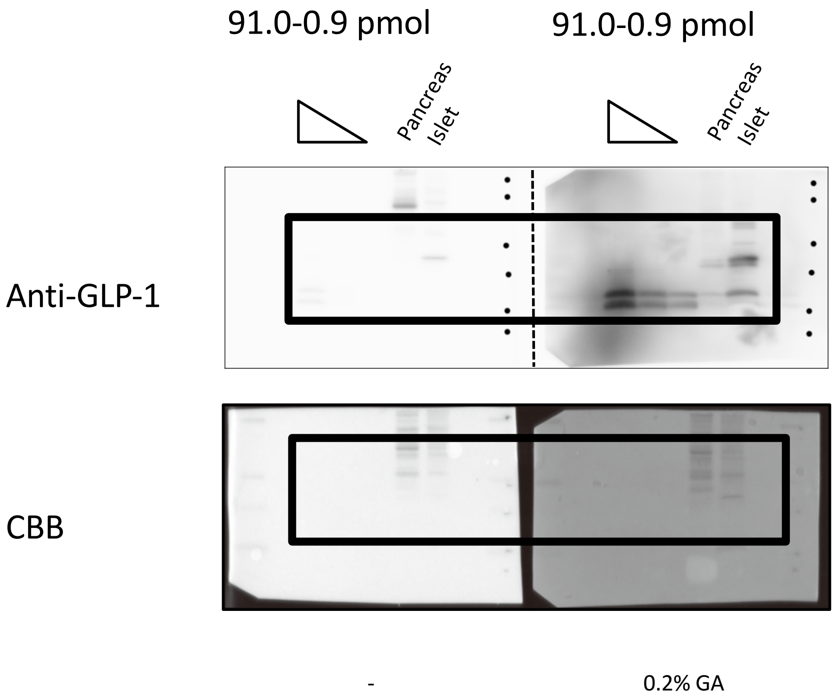

Supplement: Supplementary file 1 — Supplemental Figures [file 41598_2017_4456_MOESM1_ESM.pdf]
